# Ligand-Linked Nanoparticles-Based Hydrogen Gas Sensor with Excellent Homogeneous Temperature Field and a Comparative Stability Evaluation of Different Ligand-Linked Catalysts †

**DOI:** 10.3390/s19051205

**Published:** 2019-03-09

**Authors:** Anmona Shabnam Pranti, Daniel Loof, Sebastian Kunz, Volkmar Zielasek, Marcus Bäumer, Walter Lang

**Affiliations:** 1 IMSAS—Institute for Microsensors, -actuators and -systems, University of Bremen, 28359 Bremen, Germany; wlang@imsas.uni-bremen.de; 2 IAPC—Institute of Applied and Physical Chemistry, University of Bremen, 28359 Bremen, Germany; daniel.loof@uni-bremen.de (D.L.); sebkunz@uni-bremen.de (S.K.); zielasek@uni-bremen.de (V.Z.); mbaeumer@uni-bremen.de (M.B.)

**Keywords:** ligand, homogeneous temperature, nanoparticles, catalyst, stability, thermoelectric, hydrogen, combustion

## Abstract

This paper presents a thermoelectric gas microsensor with improved stability where platinum nanoparticles linked by bifunctional ligands are used as a catalyst. The sensor design provides a homogeneous temperature field over the membrane, an important factor for the long-term stability of the catalyst. A comprehensive study of heat transfer from the chip is performed to evaluate the convection heat loss coefficient and to understand its effect on the homogeneity of the temperature field in a real-time situation. The effect of highly heat-conductive thermopiles is also analyzed by comparing the temperature distribution and power consumption with a thermoresistive sensor of the same dimensions and materials. Despite the thermopiles, the thermoelectric sensor gives better temperature homogeneity and consumes 23% less power than the thermoresistive sensor for 90 °C average temperature on the membrane. A comparative stability analysis among ligand-linked nanoparticles with 5 different ligands and unprotected nanoparticles was done through 3 consecutive 24 h tests under 1.5% continuous hydrogen gas flow. The sensors give very stable output, almost no degradation, through 72 h (3 × 24 h) tests for 3 different ligand-linked nanoparticles. The sensor design provides superb stability to the catalyst: Even catalysts of unprotected nanoparticles withstood more than 24 h and the sensor signal degradation is only 20%.

## 1. Introduction

Hydrogen, an important gas for a wide range of scientific and industrial processes and one of the most promising renewable energy carriers for the near future, has a large explosive range (4–75%) [1,2,3,4,5]. Although hydrogen detection and concentration measurement technologies were implemented more than 100 years ago, still now precision, robustness and stability of the detection and measurement technologies and devices are the key issues for industrial leakage detection as well as for safe production, transportation and handling of hydrogen as a fuel [1,3,5,6,7]. Recent developments of micro-electro-mechanical systems (MEMS) have heightened the need for the improvement and optimization of sensing materials for miniaturized gas sensors. Recently, researchers have shown an increased interest in low concentration hydrogen detection on the ppm level with increased sensitivity and low power consumption; much less attention however has been paid to the robustness and the long-term stability of the sensors [5,8]. The most significant current discussions in hydrogen gas sensing technology (around 40% of the current research of the field) are the thermal conductivity sensor and the semiconductor metal oxide sensor although the signal of these sensors varies with environmental temperature and saturates at high gas concentration, and the sensors have high cross-sensitivity to other reducing gases [1,3,6,9]. In contrast, catalytic combustion sensing introduced in 1923 by Jonson is a traditional and effective technique of selective hydrogen detection as a considerable amount of heat (141.9 kJ/g) is released during the exothermic hydrogen oxidation [1,3]. The use of catalysts such as platinum or palladium helps to enhance the reaction rate at a lower temperature by providing bond breaking pathways. Both, the thermoelectric and the thermoresistive transduction principle are used to convert the generated heat due to the hydrogen combustion into an electrical signal [1,3]. Since first reported in 1985 by McAleer, a number of studies on thermoelectric catalytic gas-sensing were performed in the last decades with catalysts such as thin films as well as porous films of Pt or Pd on ceramic supports that consume comparatively high power and show low sensitivity [3]. For instance, the catalytic thermoelectric sensors reported in the last decade by Qui et al., Tajima et al., Shin et al. and Matsumiya et al. provided 3.75, 6.5, 9.4 and 8.5 mV output, respectively, for a 3% hydrogen gas concentration [2,8,10,11]. It was only after a while, that Sturm et al. reported about a thermoelectric combustible gas sensor again. It showed improved sensitivity as sputtered nonporous platinum thin films were used as catalyst [12]. More recently, researchers have started using catalysts made from metal nanoparticles and thereby initiated a new era in gas sensing technology with high sensitivity and low power consumption [13,14]. Au nanoparticles protected by alkanethiol were used as a catalyst by Wohltjen and Snow in 1998 [13]. Garg et al. prepared a volatile organic compound (VOCs) sensor with thiol monolayer-coated Au nanoparticles. Although the sensor had high sensitivity, long-term stability was the main problem associated with thiol-capped Au nanoparticles, and monothiol-gold bonds degraded over time [13]. Ju et al. investigated single-walled carbon nanotubes (SWCNTs) grafted with Pd nanoparticles, Haija et al. used CuFe_2_O_4_ nanoparticle based thin film and a thermoresistive sensor with highly crystalline TiO_2_ nanorods was presented by Singh et al. for hydrogen gas sensing [15,16,17]. These sensors showed high sensitivity, however, the stability of the sensors was not investigated. Sadek et al. presented a thermoresistive sensor with polyaniline nanofibers. The sensor was tested at 1% hydrogen and instability was found in the sensor signal [18]. Kabcum et al. found 25% and Wang et al. found 10% signal drops during the long-term stability test of their hydrogen gas sensors based on PdO nanoparticle-decorated WO_3_ nanorods and Au-loaded SnO_2_ composite nanoparticles, respectively [19,20]. Altmann et al. revealed a new possibility of highly sensitive thermoelectric catalytic gas sensing (6.4 mV/0.1%) with Pt nanoparticles stabilized by dodecylamine (DDA) as a ligand [21]. Recently published literature on the catalytic combustible sensor by Lee et al. and Harley-Trochimczyk et al. have only highlighted the low power consumption of the sensor without commenting on the stability of the catalyst [22,23]. Most of the studies in the field of nanoparticles-based combustible gas sensors have only focused on the improvement of sensitivity and reduction of power consumption of the sensor. No previous study has investigated and paid attention to the homogeneity of the temperature distribution on the catalyst and evaluated the power consumption based thereon, with low power consumption being recognized as one of the most important requirements for the long-term stability of the nanoparticles-based catalyst [24,25,26]. In an inhomogeneous temperature field, the nanoparticles on the higher temperature region may sinter and the nanoparticles on the lower temperature region may become inactive due to water accumulation on the layer [24]. Both sintering and water accumulation lead to the reduction of sensor sensitivity and stability. Therefore, not only low power consumption but also homogeneous temperature distribution are among the most important factors that ensure long-term stable operation of nanoparticles-based catalytic gas sensors.

We present here a thermoelectric catalytic hydrogen gas sensor with an excellent homogeneous temperature distribution on the membrane and 23 mW power consumption at 110 °C operating temperature. A much debatable question for this specific design of the sensor is to what extent the highly heat conductive thermopile contributes to the power consumption and the performance of the sensor. A reliable comparative evaluation of the temperature distribution on the membrane of the sensor with and without thermopile is necessary for elucidating the above-mentioned question.

Previous research conducted by Brauns et al. has established that the use of ligand-stabilized Pt nanoparticles as a catalyst improves the sensitivity and power consumption of the sensor by many folds [24,27]. In this previous work hexadecylamine (HDA), diaminooctane (DAO) and *p*-phenylene-diamine (PDA) ligands were employed to link 2 nm diameter Pt nanoparticles. An extra Au layer on the membrane was used to increase the adhesion of the catalyst. There is room for improvement: the Au layer causes extra heat loss from the membrane [24,27]. Furthermore, absorption of any molecules such as water produced during hydrogen combustion can deactivate the ligand-linked nanoparticle catalyst [13,24]. These molecules may penetrate the ligand-nanoparticles network and cause swelling that ultimately damages the stability of the network [13]. Moreover, so far the stability evaluation data was limited to only one kind of ligand-nanoparticles network for 1% hydrogen concentration in the previous work [24]. Therefore, a comparative stability evaluation of different ligand-linked nanoparticles catalysts is important to select the best one for long-term stable operation of the sensor. Due to varying size and functionality, different ligands may optimize the rigidity of the ligand-nanoparticles network and its binding to the membrane (thereby eliminating the necessity of an extra Au layer for adhesion improvement), and may improve the overall stability and sensitivity of the sensor. With these targets in mind, a selection of five aromatic ring-based bifunctional ligands was done for the synthesis of Pt nanoparticle-ligand networks and deposited as catalyst onto the membrane of thermoelectric micro-gas sensors with optimized design. In the present work, we focus on the design issue of the thermoelectric gas sensor related to the stability of the catalyst and a comparative performance evaluation of the sensor for the different ligand-linked nanoparticles catalysts with respect to stability.

## 2. Materials and Methods

### 2.1. Sensor Fabrication

**Figure 1:**
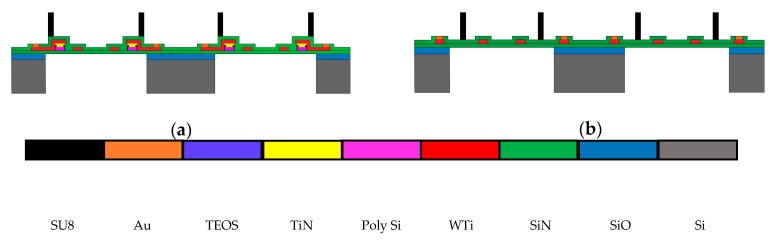
Schematics of the (**a**) thermoelectric sensor and (**b**) thermoresistive sensor.

The schematic cross section of the fabrication layers of the thermoelectric sensor is shown in Figure 1a. The sensor was fabricated on a 380 µm thick Si wafer with a 500 nm thick thermal oxide layer. This thermal oxide layer acts as an etch stop layer during the deep reactive ion etching (DRIE) of Si wafer for creating the membranes. High temperature-stable and low-stress silicon nitride with a tensile stress of 134 MPa was used as a membrane material and that was deposited in two individual 300 nm thick layers by two low-pressure chemical vapour deposition (LPCVD) processes at 800 °C. After the deposition of the lower silicon nitride layer (300 nm), a 300 nm p-doped polysilicon layer was formed and structured by reactive ion etching (RIE) as the first thermopile material. Afterwards, 80 nm thick silicon oxide layer was created by chemical vapour deposition (CVD) of TEOS process followed by a 60 nm thick titanium nitride deposited by reactive sputtering and structured. Silicon oxide (TEOS) was used as an etch stop layer for reactive ion etching (RIE) of the titanium nitride layer which is a diffusion barrier between two thermopile materials (polysilicon and tungsten-titanium). A 200 nm tungsten-titanium (WTi, 90% W/10% Ti) alloy was formed by sputtering and structured wet chemically for the second thermopile material and heater. After that, the upper silicon nitride (300 nm) layer was deposited as the top insulation of the membrane. Additionally, a 40 μm high cylinder was created on each membrane by spin coating of SU-8 3025 from MicroChem Corp. (Westborough, MA, USA) as a catalyst reservoir. A thick gold layer (300 nm) was used on the contact points of the heater and the thermopiles as bond pads. Finally, the membranes were released by DRIE and the sensors were separated by sawing. The fabrication process is based on the process described by Buchner et al. [28].

In order to assess whether and how the sensor design with thermopile is advantageous based on temperature distribution and power consumption, a thermoresistive sensor was fabricated with the same dimensions and the materials and on the same wafer so that both sensors face the same condition during fabrication processes and a perfect comparative evaluation of their performance can be done. A schematic cross-section of the fabrication layers of the thermoresistive sensor is shown in Figure 1b.

### 2.2. Preparation of Catalytic Layers

**Figure 2:**
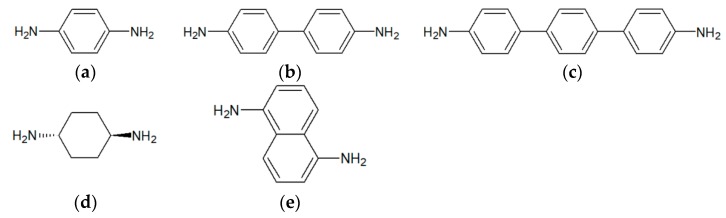
Chemical structures of bi-functional ligands: (**a**) *p*-phenylenediamine (PDA), (**b**) benzidine (BEN), (**c**) 4,4′′-diamino-*p*-terphenyl (DATER), (**d**) *trans*-1,4-diaminocyclohexane (DACH), (**e**) 1,5-diaminonaphthalene (DAN).

Pt nanoparticles linked by bi-functional ligands were used as a catalytic layer for the sensors. Stability is a classical problem of catalytic micro gas sensors and the ligand is an important component that plays a key role in the stability of the catalyst. Five different ligands with two amino head groups were used in this work for creating a ligand-nanoparticles network. Figure 2 shows the chemical structures of the different ligands used in this work.

A polyol synthesis process was used to prepare unprotected Pt nanoparticles. For this purpose, 0.5 g hexachloroplatinic acid (H2PtCl6 × H_2_O) in 50 mL alkaline ethylene glycol (0.25 M NaOH) was used whereby the Pt concentration was 4.0 g/L (20.5 mM). The process was performed at 160 °C for 1.5 h. The clear yellow precursor solution was turned into black when Pt nanoparticles colloid was formed. Further cleaning and redispersion of the Pt nanoparticles were done before linking with ligands. For precipitation, 6 mL of 1 M aqueous HCl solution was added into 3 mL of Pt colloid solution. The suspension was centrifuged and the supernatant solution was removed. This process was repeated one more time. The precipitated Pt nanoparticles were redispersed in 0.5 mL of cyclohexanone resulting in the black colloid of 24 g/L Pt (123 mM).

Always freshly prepared unprotected Pt nanoparticles were used to link with ligands for higher quality linkage. Cyclohexanone was used as a solvent to prepare all the ligand solutions. As we applied an equimolar ratio of Pt nanoparticles and ligand, equal volumes of 123 mM ligand (PDA, BEN, DACH, DAN) and 123 mM Pt nanoparticles colloid were mixed with each other to form the catalytic layer. The only exception is DATER since it is less soluble. Here, a volume of 123 mM Pt nanoparticles solution was mixed with a 50% larger volume of 82 mM DATER solution. A list of all chemicals, used as purchased for catalyst preparation, along with their weight % or purity and suppliers is presented in Table 1.

### 2.3. Catalyst Deposition on the Membrane of the Sensor

Ligands and nanoparticles were directly linked on the membrane of the sensor very precisely in a systematic way. For that purpose, a micro dispenser from Microdrop Technologies GmbH (Norderstedt, Germany) was used that contains two distinct nozzles (MD-K-130) and two control units (MD-E-3010). The ligand and the nanoparticle solution were applied on the membrane by the two distinct nozzles consecutively. The catalytic layer deposition process will be presented in detail in a forthcoming paper.

### 2.4. Working Principle of the Sensors

The temperature of the active area (hot end of the thermocouple) of a thermoelectric sensor increases due to the exothermic heat of hydrogen combustion, which depends on the concentration of the reacted gas. A temperature difference is created between the hot and the cold end of the thermocouple and thereby a thermoelectric voltage is induced according to the following formula [3]:

U = α·ΔT(1)

Here, the sensor signal U depends not only on the temperature increment ΔT due to the hydrogen combustion but also on the Seebeck coefficient (α) of the thermocouple material of the sensor. ΔT should be proportional to the molar reaction enthalpy of hydrogen combustion reaction and the hydrogen concentration (in a synthetic air flow) [3]. 

**Figure 3:**
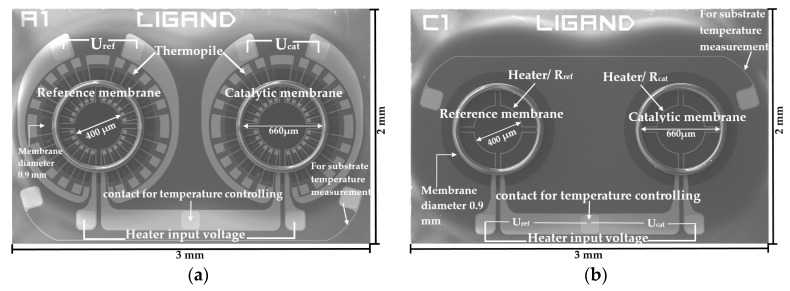
SEM image of the top view of (**a**) the thermoelectric sensor (sensor output = U_cat_ − U_ref_) and (**b**) thermoresistive sensor (sensor output is ΔR_cat_ = ΔU_cat_/I_s_ where I_s_ is the current flows through the heater).

Figure 3a,b shows scanning electron microscopy (SEM) images of the top view of the thermoelectric and the thermoresistive sensor, respectively. Both membranes of the thermoelectric sensor and the thermoresistive sensor are heated to their operating temperature by supplying a necessary constant voltage to the heater. The heat released by the exothermic reaction of hydrogen combustion on the catalytic membrane increases the temperature of the active area of the membrane, with the increment being proportional to the concentration of hydrogen gas. In the thermoelectric sensor (Figure 3a), the thermopile of the catalytic membrane generates a voltage (U_cat_) that is also proportional to the increment of the temperature due to the hydrogen combustion reaction. On the other hand, the thermopile of the reference membrane produces a voltage (U_ref_) if any change in the environment occurs. The voltage generated by each thermopile is 6.55 mV/K with a Seebeck coefficient of 273 µV/K. Therefore, the final output of the thermoelectric sensor is the difference between the catalytic thermopile and the reference thermopile voltages (sensor output = U_cat_ − U_ref_). In case of the thermoresistive sensor (Figure 3b), the WTi heater is used not only for heating the catalyst but also for sensing the change of the temperature due to the exothermic reaction of hydrogen combustion. The change in voltage drop across the catalytic heater is measured during the combustion reaction (ΔU_cat_) to calculate the change of the heater resistance. Additionally, a small constant resistance (R_s_) is used in series with a heater to measure the current through the heater during the reaction (I_s_). Therefore the change in catalytic heater resistance is ΔR_cat_ = ΔU_cat_/I_s_. The change in the resistance of the reference membrane is calculated similarly and the function of the reference membrane is the same as the function of the reference membrane of the thermoelectric sensor. Provided that, the thermoresistive sensor was only used for a comparative analysis of the thermal performance of the sensor in this study.

### 2.5. Measurement Processes

The temperature distribution on the membrane was simulated by using the COMSOL Multiphysics software before the fabrication of the sensor. Additionally, the real temperature distribution on the membranes of the sensor was examined after the fabrication of the chip with an infrared (IR) camera. The analysis was done for different average temperatures of the membrane by applying necessary constant DC voltage. The measurement was done in a dark room and with a black box covering the camera for protecting outside light to avoid reflection from the surface of the sensor. IRBIS plus software, version 2.2 was used for analyzing the temperature profile and the average temperature on the membrane for different constant DC voltages applied to the heater.

When hydrogen combustion proceeds for a certain time, due to the continuous heat generation by the exothermic reaction the temperature of the heater as well as the catalytic layer increases beyond the operating temperature. Ligands may become unstable at very high temperature that can lead to sintering among the nanoparticles [24]. As a result, the catalytic layer is damaged and the sensitivity of the sensor reduces. To avoid this phenomenon, the temperature of the heater of the catalytic membrane was controlled by using the temperature coefficient of resistance (TCR) of the heater material for all long-duration measurements (more than 10 min). A simple analogue circuit using an instrumentational amplifier, LT 1167, was used for this purpose instead of microcontroller-based controlling circuit because it was observed from our experiments that the microcontroller based controlling system is at least 40% slower than the simple analogue controller circuit. The controller gain was calibrated before each experiment at different temperature and with different chip for precise controlling.

### 2.6. Gas Measurement Setup

**Figure 4:**
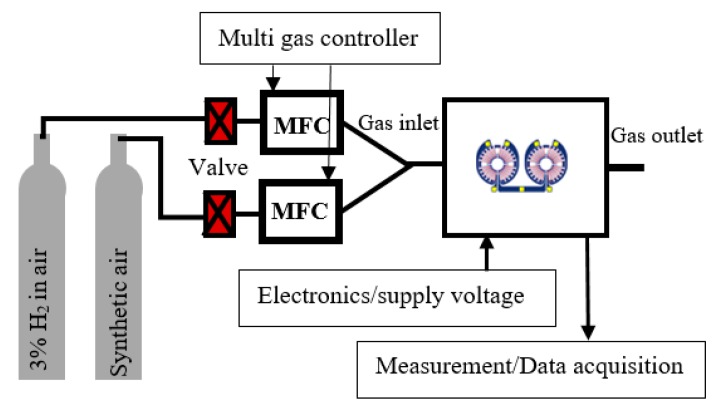
Schematic of the gas measurement setup.

Figure 4 shows a schematic of the gas measurement setup. 3% hydrogen in synthetic air from Air Liquide (Krefeld, Germany) was used for the experiments as the lower explosive limit (LEL) of hydrogen is 4% [2]. 3% hydrogen was further mixed with synthetic air from Air Liquide, Kornwestheim, Germany to obtain the desired concentration. Two mass flow controllers from MKS Instruments (Munich, Germany), type 1179B, were used to control the flow rate of hydrogen and synthetic air and the mass flow controllers were controlled by a multichannel flow ration/pressure controller from MKS Instruments, type 647C. Although the gas correction factor for hydrogen and air were set in the multi-channel flow ration/pressure controller, the flow rate was crosschecked before each experiment by an electronic model 5067-0223 flow meter from Agilent Technologies (Waldbronn, Germany). The sensor was bonded on a PCB connector by aluminium wire bonding and was kept inside a PTFE experimental housing of 1.3 mL volume with an inlet and outlet. The total flow rate that entered into the experimental housing, after mixing 3% hydrogen and synthetic air, was 40 sccm. The total flow rate was kept constant for all the experiments regardless of the concentration of the hydrogen gas. The electronics, controller and supply voltage were connected to the PCB from outside of the housing. A LabVIEW program was used to acquire measurement data by using a model NI USB-621216 input channel data acquisition device from National Instruments (Austin, TX, USA).

## 3. Results and Discussion

**Figure 5:**
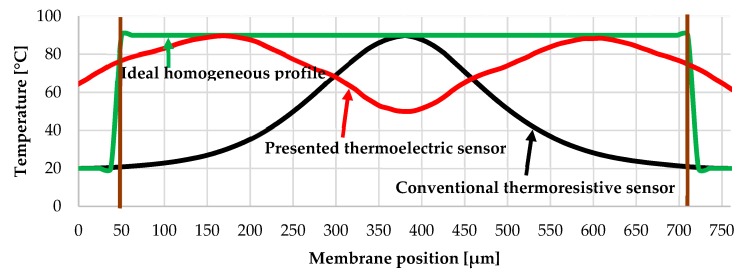
Comparison of the temperature profile (along x-axis through the diameter, 0 µm position starts from the outer periphery of SU8 ring shown in Figure 3a); presented thermoelectric sensor, conventional thermoresistive sensor and ideal homogeneous temperature profile (the red curve is better than the black curve).

The sensor is designed to have a homogeneous temperature distribution over the membrane for improving the stability of ligand-linked nanoparticles. The round shape membrane and the circular heater of the sensor give more homogeneous temperature distribution than a conventional square or rectangular membrane like references [23,24,26], we found it through the simulation in COMSOL Multiphysics software. Figure 5 shows the temperature profile of an ideal homogeneous distribution, a conventional resistive sensor with a rectangular membrane (simulated) and the real temperature distribution of our thermoelectric sensor (measured by IR camera). The main idea behind the sensor design was to have a perfect homogeneous temperature profile like the green curve. However, the sensor also had to fulfil some other requirements. For an example, the hot end of the thermopile was placed very close to the heater so that the hot end temperature becomes almost the same as the heater temperature. The hot end and the cold end of the thermopile were placed away from each other, as much as possible, so that the temperature difference between them remained as high as possible. The sensor was designed to fulfill all those requirements and hence a perfect ideal homogeneous temperature field as the green curve was not achieved (red curve of Figure 5). However, the temperature profile of our thermoelectric sensor is far better and more homogeneous than the temperature profile of the conventional thermoresistive sensor (black curve of Figure 5) where the catalyst at the edge of the membrane will not be heated at all and the catalyst on the middle of the membrane will face high temperature that can lead to the sintering of the nanoparticles.

The area of interest for the catalyst, inside the SU8 ring (Figure 3), is defined by the brown straight lines in both sides of the curve in Figure 5. The root-mean-square deviation (RMSD) from the ideal value (green curve) is calculated for the presented thermoelectric sensor (red curve) and the conventional thermoresistive sensor (black curve) within the area of interest as shown in Figure 5 according to the following formula [29]:

(2) $$\mathrm{RMSD}=\sqrt{\frac{\sum_{i=1}^{n}d_{i}{}^{2}}{n}}=\sqrt{\frac{\sum_{i=1}^{n}{\left(\mathrm{measured}\;\mathrm{or}\;\mathrm{simulated}\;\mathrm{value}\;-\mathrm{ideal}\;\left(\mathrm{constant}\right)\;\mathrm{value}\right)}^{2}}{n}}$$

The presented thermoelectric sensor (red curve) has a root-mean-square deviation (RMSD) of 18.86 °C whereas the conventional thermoresistive sensor (black curve) has a root-mean-square deviation (RMSD) of 44.55 °C than that of the ideal value (green curve).

**Figure 6:**
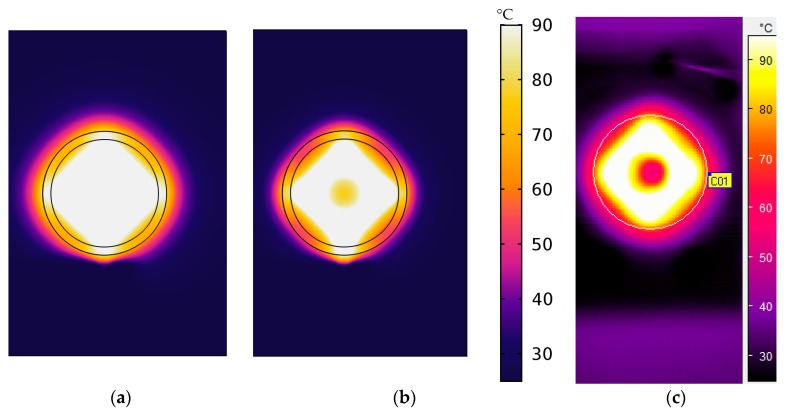
Temperature field on the membrane for 90 °C average temperature (**a**) and (**b**) from simulation with convection heat loss coefficient of 100 W/m^2^ K and 217 W/m^2^ K respectively (**c**) real measurement with IR camera for the thermoelectric sensor; (**a**) is more homogeneous than (**b**,**c**) where the small low-temperature circle may be caused by high convection heat loss.

Figure 6a,b shows the temperature field for an average 90 °C temperature on the membrane of a model of the sensor that was simulated in COMSOL Multiphysics software with a convection heat loss coefficient of 100 W/m^2^ K and 217 W/m^2^ K, respectively. The colour legend is the same for Figure 6a,b. Figure 6c shows the real temperature field for an average 90 °C temperature on the membrane by IR camera measurement. A profound small circle of low temperature is seen in the middle of the membrane in both Figure 6b,c that may be due to the high convection heat loss coefficient for a small chip area. The circle is not found in Figure 6a and the temperature distribution is more homogeneous that was simulated with a lower convection heat loss coefficient, 100 W/m^2^ K. It can thus be suggested that the convection heat loss from the membrane plays a profound role in the homogeneity of the temperature field. A possible explanation for some dissimilarities between simulated temperature distribution at 217 W/m^2^ K convection heat loss coefficient (Figure 6b) and real measurement (Figure 6c) might be that the considerations for simplifying the simulation such as a standing air is only considered inside the active area of the membrane but the real situation is slightly different.

**Figure 7:**
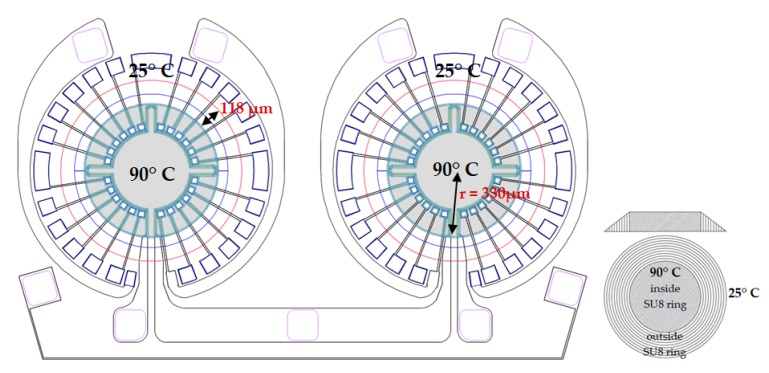
Criteria of the heat loss modelling.

To support the above-stated statement that the low-temperature circle in the middle (from real measurement in Figure 6c) is created due to the high convection heat loss coefficient for a small chip, a mathematical calculation of the entire heat transfer from the chip along with the convection heat loss coefficient is done. The purpose of this calculation is to compare the calculated power loss with the real power measurement to verify the convection heat loss coefficient that is mathematically calculated and considered for COMSOL Multiphysics simulation (Figure 6b). Heat transfer from the chip can occur in three ways, by conduction through the thermopile elements and membrane material, by convection to the surrounding from the up and downside of the membrane and through radiation. Heat loss through radiation is neglected in the mathematical calculation as this is very small compared to the conduction and the convection heat loss as the thermal emissivity of silicon nitride thin film is approximately 0.3 [30,31,32]. Only in-plane conduction heat transfer is taken into account during calculation as the cross-plane conduction heat transfer is negligible for the chip with low thickness layer and membrane [31]. It is considered that the average temperature in the active area (inside the SU8 ring) is 90 °C as shown in Figure 7 and conduction heat loss occurs through the thermopile materials (WTi and polysilicon) and membrane material (SiN) from inside periphery of the SU8 ring to the end of the membrane where the temperature is considered as 25 °C (Figure 7). Power loss through in-plane heat conduction in case of DC joule heating can be explained by the one-dimensional heat transfer equation and is completely linear with temperature increment [30,31,32,33]:

K = (Q·L)/(b·h·ΔT)(3)

Here Q is the transferred heat flux through conduction that is denoted latter as P_cond_ throughout the text, L, b and h are the length, width and thickness of the film respectively through which heat conduction loss occurs and K is conduction heat loss coefficient of the respective material. ΔT represents the temperature difference between the places where power loss occurs and is 65 °C for our case. The number of the thermocouple on each membrane of the chip is n = 24. The conduction heat loss coefficient of tungsten-titanium (K_WTi_), polysilicon (K_poly_) and silicon nitride (K_SiN_) are 158.8 W/mK [34], 60 W/mK [35] and 3.99 W/mK [36] respectively.

Conduction heat loss due to the thermopile material (WTi):

P_cond(WTi)_ = 2·n·(K_WTi_·(b_WTi_·h_WTi_)·(ΔT/L_WTi_))            = 2·24·(158.8 W/mK·(8 µm·0.2 µm)·(65 °C/118 µm)) = 6.8 mW

Conduction heat loss due to the thermopile material (polysilicon):

P_cond(poly)_ = 2·n·(K_poly_·(b_poly_·h_poly_)·(ΔT/L_poly_))            = 2·24·(60 W/ mK·(12 µm·0.3 µm)·(65 °C/118 µm)) = 5.6 mW

Conduction heat loss due to the membrane material (silicon nitride):

P_cond(SiN)_ = K_SiN_·(b_SiN(inner periphery of SU8 ring)_·h_SiN_)·(ΔT/L_SiN_)          = 3.99 W/mK·(2073.5 µm·0.6 µm)·(65 °C/118 µm) = 5.4 mW

The heat flux loss (q, later denoted as P_convec_) from a heated surface area A to the surrounding fluid can be expressed by the following equation called Newton’s law of cooling [30]:

q = α_conv_·A·ΔT (4)

Here, α_conv_ is the convection heat loss coefficient which can be calculated with the help of a dimensionless number Nu called Nusselt number and ΔT is the temperature difference between the heated surface and the fluid [30]:

α_conv_ = (Nu·K_fluid_)/l_cha_ (5)

Here K_fluid_ is the thermal conductivity of the surrounding fluid and l_cha_ is the characteristic length. Since the Nusselt number is a function of the other two dimensionless number called Prandtl number (Pr) and Graßhoff number (Gr) can be calculated by the following equation [30]:

Nu = Nu_0_ + 0.6·Gr^1/4^·Pr^1/3^ (6)

Considering spherical behaviour of the heat transfer system with a diameter equal to the characteristic length, one can obtain Nu_0_ (sphere, ΔT = 0) = 2. Pr is a constant value for a specific fluid and can be obtained by dividing the thermal diffusivity by the viscosity of the fluid. On the other hand, Gr represents the ratio of the bouncy to the viscous force and can be obtained by the following formula [30]:

Gr = (l_cha_^3^·g·β·ΔT)/ν^2^ (7)

Here β and ν are the thermal expansion and the viscosity of the fluid and g is the acceleration by gravity.

The characteristic length of the problem is calculated as follows [30]:

l_cha_ = 0.5·0.625·(diameter of the membrane + width of the heater) = 285 µm

Here membrane diameter = 896 µm and the heater width = 16 µm:

Gr = (l_cha_^3^·g·β_air_·ΔT)/ν_air_^2^ = 0.202

Here g = 9.81 m/s^2^, thermal expansion of air, β_air_ = 3.363 × 10^−3^/K [37], Viscosity of air, ν_air_ = 15.78 × 10^−6^ m^2^/s [37] at 25 °C temperature.

The Prandl number of air is considered Pr = 0.7 at 25 °C [30], hence Nu = 2.3570.

Convection heat loss coefficient with the value of thermal conductivity of air, K_air_ = 0.02625 W/mK [37] at 25 °C is α_conv_ = (Nu·K_air_)/l_cha_ = 217 W/m^2^ K.

It is considered that the temperature outside the active area (grey area in Figure 7) decreases gradually from 90 °C to 25 °C. Therefore, half of the area of the membrane outside the active area is taken into account along with the active area for calculating the convection heat loss from the membrane. The area for the convection heat loss calculation A_convc_ = 0.475 mm^2^.

The convection heat loss, P_convc_ = 2·(P_convec(upside of the membrane)_ + P_convc(down side of the membrane)_) = 26.8 mW.

Total power loss, P_total_ = P_cond (WTi)_ + P_cond (poly)_ + P_cond(SiN)_ + P_convc_ = 44.6 mW

**Figure 8:**
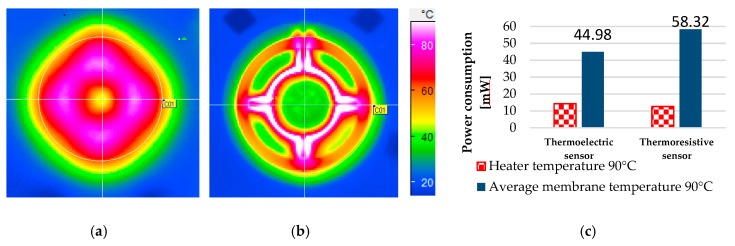
Temperature field on the membrane with IR camera measurement (**a**) thermoelectric sensor; (**b**) thermoresistive sensor, (**a**) is more homogeneous than (**b**); (**c**) comparative power consumption of the thermoelectric and the thermoresistive sensor [38].

The sensor design with highly heat conductive thermopile material encircling the round membrane helps to create a homogeneous temperature field on the membrane. A comparative performance analysis between the thermoelectric sensor and a thermoresistive sensor fabricated with the same material as mentioned in the Section 2 (materials and methods) was performed in terms of power consumption, heater temperature and the average membrane temperature. Figure 8a,b shows the temperature field on the membrane of the thermoelectric sensor and thermoresistive sensor respectively for a maximum temperature of 90 °C (colour legend same for both Figure 8a,b) [38]. It is evident from Figure 8a,b that the temperature distribution over the membrane of the thermoelectric sensor is more homogeneous than the thermoresistive sensor. Therefore, the catalyst over the membrane of the thermoelectric sensor is heated more homogeneously. Although the highly heat conductive thermopile causes high power loss, it helps to increase the homogeneity of the temperature distribution when the thermocouples are placed evenly encircling the round membrane. If we consider a specific heater temperature, e.g., 90 °C, the thermoelectric sensor consumes 14 mW and the thermoresistive sensor consumes 12.6 mW power [38]. Therefore, the power consumption of the thermoresistive sensor is only 1.6 mW lower than the thermoelectric sensor for a specific heater temperature of 90 °C. Interestingly, the thermoelectric sensor consumes even lower power than the thermoresistive sensor for an average temperature on the membrane as the thermopile provides more homogeneous temperature distribution. The thermoelectric sensor consumes 23% (13.3 mW) less power than the thermoresistive sensor for maintaining an average temperature of 90 °C on the membrane [38]. What is interesting here is that despite the fact of highly heat conductive thermopile; the thermoelectric sensor performs better than the thermoresistive sensor regarding an average temperature over the membrane. The comparison of power consumption between the thermoelectric sensor and the thermoresistive sensor for 90 °C heater temperature and 90 °C average temperature on the membrane is shown in Figure 8c. The measured power consumption of the thermoelectric sensor for 90 °C average temperature on the membrane is 44.98 mW. The most important observation is that the measured power consumption is equal to the calculated power consumption. These results corroborate the value of the calculated convection heat loss coefficient, 217 W/m^2^ K. This study confirms that the small low-temperature circle in the middle, the cause of inhomogeneity in the temperature field (Figure 6b,c and Figure 8a), is caused by high convection heat loss from the membrane. If we now focus on the above-stated formulas of Equations (4)–(7), the convection heat loss (q) as well as the Graßhoff number (Gr) followed by the Nusselt number (Nu) and convection heat loss coefficient (α_conv_) decrease when ΔT (temperature difference between the heated membrane and the surrounding fluid) decreases. Although it is very difficult to explain the change in convection heat loss phenomenon during combustion reaction, it might be reduced as the environmental temperature increases. This reduction of convection heat loss possibly causes a more homogeneous temperature field over the membrane of the thermoelectric sensor (may eliminate the small temperature circle in the middle of the membrane of Figure 6c and Figure 8a) during the operation of the sensor in hydrogen gas.

**Figure 9:**
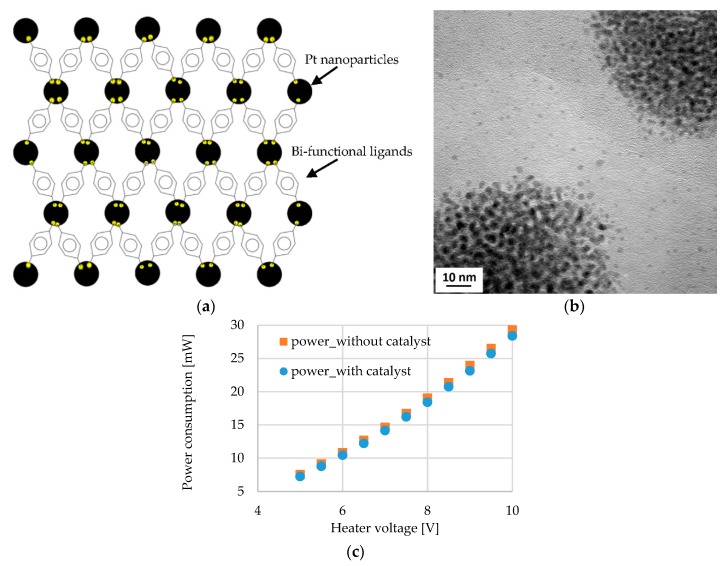
(**a**) Schematic of the nanoparticles network linked by bi-functional ligands (**b**) the transmission electron microscopy (TEM) micrograph of ligands-nanoparticles network (Pt-PDA), the spacing between the neighboring nanoparticles due to the ligands is observed (**c**) Power consumption of the sensor with the supply voltage to the heater (without hydrogen gas), the power consumption of the sensor with and without a catalyst is almost same for each supply voltage; ligand-linked nanoparticles do not contribute to the power consumption.

The idea of stabilizing nanoparticles by organic ligands is to create a ligands-nanoparticles network with free spaces [24,27] as shown in Figure 9a. These free spaces not only help to increase the sensitivity of the sensor by facilitating the diffusion of hydrogen molecules but also reduce the power consumption of the sensor [24,27]. Mono-functional ligands containing one amine group, such as hexadecylamine (HDA) can create a strong bonding with only one nanoparticle. The other tail sides of the ligands form weak van-der-Waals bonds with one another that can break quite easily [27]. In this work, five different bi-functional ligands, *p*-phenylenediamine (PDA), benzidine (BEN), 4,4′′-diamino-*p*-terphenyl (DATER), *trans*-1,4-diaminocyclohexane (DACH) and 1,5-diamino-naphthalene (DAN), were used to produce ligand-linked nanoparticle networks. The main idea of bi-functional ligands is that they contain two amino head groups that can bond strongly bonding with two different nanoparticles and thus create a strong network of the catalyst as shown schematically in Figure 9a (small yellow circles represent amino groups). A detailed characterization of the generated Pt nanoparticle networks will be provided in a forthcoming publication. Transmission electron microscopy (TEM) was performed to analyze the structure of the Ligands-nanoparticles network. A FEI Tecnai G2 F20 S-TWIN instrument (FEI company (Thermo Fisher Scientific), Hillsboro, OR, USA), operated at 200 keV electron energy was used for TEM. Samples were prepared by subsequently dispensing the ligands in solution and the nanoparticles dispersion on a carbon-coated Cu TEM grid and letting the samples to dry in the air.

The TEM micrograph of Figure 9b shows individual Pt nanoparticles within three-dimensional aggregates formed by the PDA-linked network on the TEM grid and reveals spacing between neighboring nanoparticles, as expected due to the presence of the ligands. The TEM micrograph for only Pt-PDA network is provided here, TEM micrographs for other ligands-nanoparticles network also showed similar result. Figure 9c shows the power consumption of the sensor without the catalytic layer and with a catalytic layer for different voltages applied to the heater of the sensor. Different applied voltage corresponds to different operating temperatures, e.g., 7 V and 9 V correspond to 90 °C and 110 °C operating temperature, respectively. Operating temperature means the heater temperature, not the average temperature of the membrane throughout the text. The most important observation from Figure 9c is that the power consumption of the sensor with and without a catalyst is almost the same for the different applied voltage that means for different operating temperatures. So the catalytic layer of ligand-linked nanoparticles does not contribute to the power consumption of the sensor. Figure 9c shows the curve of the power consumption depending on the applied heater voltage for only one type of ligand-linked nanoparticles, *p*-Phenylene diamine (PDA), the other 4 types of ligand-linked nanoparticles also show a similar result that is not presented.

**Figure 10:**
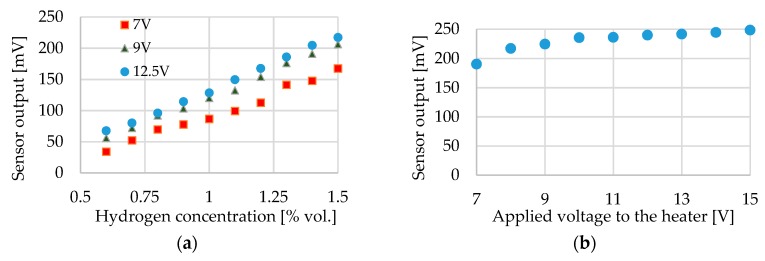
(**a**) Linear characteristics of the sensor with hydrogen concentration for different supply voltage (**b**) sensor output at 1.5% hydrogen concentration for different supply voltages; output saturates after 11 V.

The activity of the sensor was tested for three supply voltages by varying the concentration of hydrogen gas gradually from 0.5% to 1.5%. Figure 10a shows that the sensor signal increases linearly with the concentration of hydrogen for all the three supply voltages means for three operating temperatures, 7 V, 9 V and 12.5 V correspond to the operating temperatures of 90 °C, 110 °C and 130 °C, respectively. The output of the sensor for a specific gas concentration also increases with the heater supply voltage (operating temperature) up to a point and then saturates [2,9]. Increasing supply voltage (operating temperature) beyond this point increases the power consumption of the sensor unnecessarily and also can lead to sintering of the nanoparticles. Therefore for fixing the highest safe operating voltage (operating temperature), the sensor was tested for different supply voltages at a constant 1.5% hydrogen concentration. The graph in Figure 10b shows a steady increase in sensor output for the heater supply voltage up to 11 V, which becomes stable at higher voltages. The presented curve is only for PDA-linked Pt nanoparticles. The sensor shows similar results for the other four types of ligand-linked nanoparticles.

**Figure 11:**
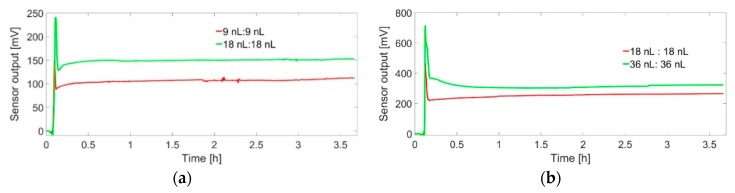
Sensor output for around 4 h at 130 °C operating temperature in continuous hydrogen flow at (**a**) 0.6% (ligand:nanoparticle = 9 nL:9 nL and 18 nL:18 nL) (**b**) 1.5% (ligand:nanoparticle = 18 nL:18 nL and 36 nL:36 nL); these curves are for Pt nanoparticles linked by PDA, influence of the amount of nanoparticles is higher for lower amount of nanoparticles that higher amount of nanoparticles.

When the amount of the catalyst is low, the sensitivity of the sensor increases with operating temperature. At the higher amount of catalyst, the sensitivity of the sensor decreases with operating temperature and the more is the amount of the catalyst; the more is the decrement of the sensitivity with an increment of operating temperature [5]. Conversely, the sensitivity of the sensor increases with the amount of nanoparticles [24]. However, the possibility of deactivation of the catalyst is also increased with the amount of catalyst that can limit the stability of the sensor. Therefore, to select a reasonable amount of catalyst, experiments with different amounts of catalyst were performed at 130 °C operating temperature for around 4 h in continuous hydrogen gas flow at different concentration. Figure 11a,b presents a comparison of sensor output at 0.6% hydrogen for the amount ratios 9 nL:9 nL and 18 nL:18 nL (ligand:nanoparticle) and at 1.5% hydrogen for the amount ratios 18 nL:18 nL and 36 nL:36 nL (ligand : nanoparticle). These ligand and nanoparticle volume ratios were achieved by applying appropriate concentrated solutions of the ligand and suspensions of Pt nanoparticles by micro dispensers. Figure 11a shows that a 100% increment of ligands/nanoparticles amount 9 nL to 18 nL increased the sensor output by 50%, although the concentration of the gas is low (0.6% hydrogen). Conversely, a 100% increment of ligands/nanoparticles from 18 nL to 36 nL increases the sensor output by only around 10%, even at higher concentration, 1.5% hydrogen (Figure 11b). Moreover, the excess amount of nanoparticles also increases the possibility of sintering. Therefore 18 nL:18 nL ligands:nanoparticles were selected for analysis of comparative stability among different catalyst. Here the amount of ligands and nanoparticles means the volume of ligand solution and nanoparticles colloid. The content of ligand and nanoparticles in the solution or colloid is provided in Section 2 (Materials and Methods). The sudden initial increases in the sensor signal in both, Figure 11a,b are due to sudden increases in the flow rate of the mass flow controller before it settled to the chosen flow rate; these do not constitute characteristics of the sensor.

**Figure 12:**
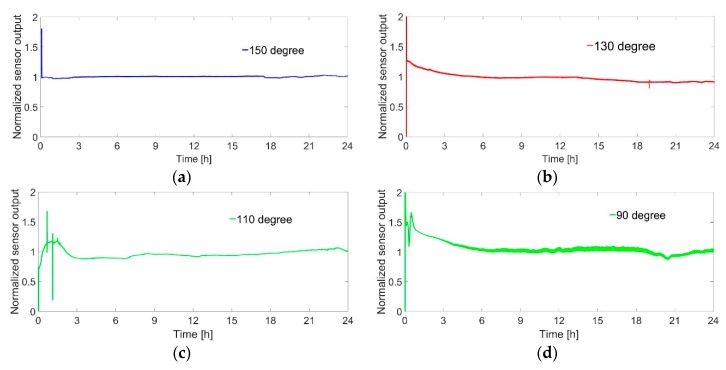
Normalized sensor output for 24 h in continuous hydrogen flow of 1.5% at operating temperature of (**a**) 150 °C; (**b**) 130 °C; (**c**) 110 °C and (**d**) 90 °C; at 150 °C, 130 °C and 110 °C stable output, output fluctuates at 90 °C.

The effect of operating temperature on the stability of the catalyst was examined by continuous hydrogen flow for 24 h at 1.5% concentration. A similar experiment with 1% hydrogen was performed by Brauns et al. for 13 h [27]. We did the experiment at a higher concentration as well as for longer time. The main motivation behind this experiment is the following: As a higher concentration of hydrogen at a higher operating temperature for a longer time can lead to the deactivation of the catalyst more easily and faster, we can better prove the improvement of the stability of the catalyst compared to the previous work done by Brauns et al. Figure 12a–d shows the normalized value of the sensor output for different operating temperature. The sensor signal remained completely stable over 24 h for higher operating temperatures such as 150 °C (Figure 12a). It indicates that the catalyst remained stable and no sintering may occur among the nanoparticles during the 24 h experimental period. The catalyst takes some time to be stabilized at a comparatively lower temperatures such as 130 °C, 110 °C and 90 °C. The time to reach the equilibrium output of the sensor is also influenced by the hydrogen concentration and flow rate, size of the housing, amounts of heat emission from the surface to the atmosphere etc. [8]. After stabilization, the sensor signal was quite stable for 24 h at 130 °C and 110 °C operating temperature (Figure 12b,c). The fluctuation in the sensor signal after 9 h at 90 °C operating temperature can be caused by the water accumulation on the catalyst (Figure 12d). Although higher temperature operation (150 °C) gives the best stable output of the sensor, higher temperature operation also causes higher power consumption.

**Figure 13:**
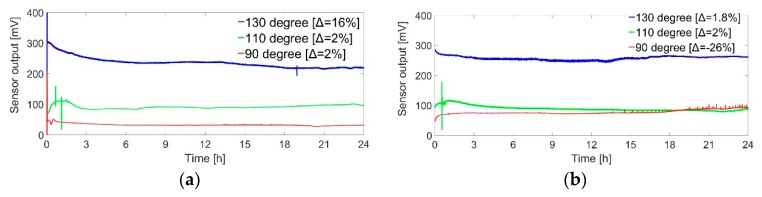

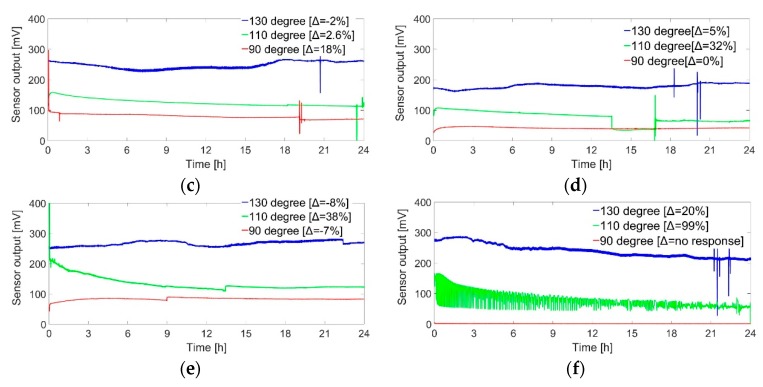
Sensor output for 72 h test (combination of three 24 h test at 130 °C, 110 °C and 90 °C operating temperature) at 1.5% continuous hydrogen flow for (**a**) Pt-PDA; (**b**) Pt-DATER; (**c**) Pt-BEN; (**d**) Pt-DAN; (**e**) Pt-DACH; (**f**) Pt nanoparticles; Δ represents the % variation in the output signal of the sensor in 24 h, (-) sign means the sensor output signal has increased due to the electrical signal fluctuation in the controller circuit, this change is not caused by the catalyst deactivation; stable output for five ligand-linked nanoparticles and only 20% reduction for non-stabilized nanoparticles at 130 °C.

A comparative stability analysis among the catalysts of Pt nanoparticles linked by five different ligands and non-stabilized nanoparticles without ligand was done. The amount of catalyst was ligand:nanoparticle = 18 nL:18 nL. The test was done in three steps for three different operating temperatures at 1.5% continuous hydrogen flow for 24 h. The 1st 24 h test was done at 130 °C operating temperature. The 2nd 24 h test was done at 110 °C operating temperature on the same sensor after 6 days of the first test. Finally, the 3rd 24 h test was done at 90 °C operating temperature on the same sensor after 6 days of the second test. Therefore, all the sensors faced altogether 72 h of 1.5% continuous hydrogen gas flow. Previous research has established that the damage of the catalytic layer that is generated at lower operating temperature is cured when the sensor is again run at higher operating temperature but the damage that is created at higher operating temperature such as at 130 °C is irreversible [24]. The main motivation and benefit of our experimental approach (starting from a higher temperature experiment at 130 °C) is that the rigidity and stability of the catalytic layer can better be proved and the irreversible damage can better be analyzed at a rugged condition. A temperature controller was used for controlling the temperature of the heater of the catalytic membrane during all the experiments. Figure 13a–f shows the output of the sensor during three 24 h tests for 6 different catalysts.

As first observation, the sensor signal remained almost constant for the 1st 24 h test at 130 °C for all the ligand-linked nanoparticles and the sensor signal with non-stabilized nanoparticles (without ligand) was reduced by around 20%. It is evident that ligand-linked nanoparticles are more stable and robust than the non-stabilized nanoparticles against sintering effect at a higher operating temperature. However, only 20% sensitivity reduction of the non-stabilized nanoparticles over 24 h operation at a high temperature is superior to previous tests and may be a benefit of the improved design of the sensor with better homogeneous temperature field. The sensitivity of the sensor with non-stabilized nanoparticles dropped by 90% in only one hour even at lower hydrogen concentration (1%), as reported by Brauns et al. in similar work [27].

As second observation, the sensors with Pt-PDA, Pt-DATER and Pt-BEN showed quite stable output during the 2nd 24 h test at 110 °C operating temperature. The signal of the sensor with Pt-DAN and Pt-DACH deviated around 35% over this 2nd 24 h test. The most striking behaviour of the sensor signal with unprotected (non-stabilized) nanoparticles was observed during the 2nd 24 h test. The sensor signal continuously fluctuated in a periodic manner with decreasing amplitude. This result may be caused by a sintering process among the nanoparticles which presumably progresses arbitrarily. Due to the build-up of mechanical stress within the catalyst layer, some particles or catalyst portions may detached from the membrane surface and reattach to some place else on the membrane. Thereby, the active area of the catalyst would change continuously. Another possible explanation for the fluctuation of the sensor signal maybe occasional peeling-off of the catalytic layer from the membrane during the sintering process. At any rate, however, the main observation from the experiment is the catalytic layer become inactive at the end of the test after a sufficient amount of sintering among the nanoparticles, the sensitivity of the sensor decreased 99% after this 2nd 24 h test.

As third observation, the sensor output was stable during the 3rd 24 h test at 90 °C for Pt-PDA and Pt-DAN. 18% sensitivity reduction for Pt-BEN can be caused by water accumulation at lower temperature operation. On the other hand, signal fluctuation and 26% increment in sensor signal for Pt-DATER may not be caused by the catalyst deactivation but due to the electrical signal fluctuation due to the controller circuit. Sensors with unprotected nanoparticles did not respond at all during the 3rd 24 h test.

The sensitivity of almost all the sensors reduced from the 1st 24 h test to the 2nd 24 h test and so on (Figure 12a–f). The 1st, 2nd and 3rd 24 h test were performed at 130 °C, 110 °C and 90 °C operating temperature, respectively. Logically, the sensor sensitivity should be lower at a lower temperature operation. Therefore, this sensitivity reduction may not only be caused by the deactivation or change of the catalyst characteristic between the 6 days of two 24 h test.

It should be noted that the sharp spikes in few curves of Figure 13 were artefacts caused when the gas flow briefly changed due to the start of parallelly running experiments since the gas lines of these experiments shared the same gas cylinder reservoir via a common main line. Sudden changes of flow in individual gas lines also changed the flow rate in other experiments until all MFC had stabilized again. This phenomenon is not related to the sensor characteristics. The sudden decrease in the curve of the experiment at 110 °C (green) in Figure 13d is observed. This is due to the failure of electrical connection of the controller circuit.

**Figure 14:**
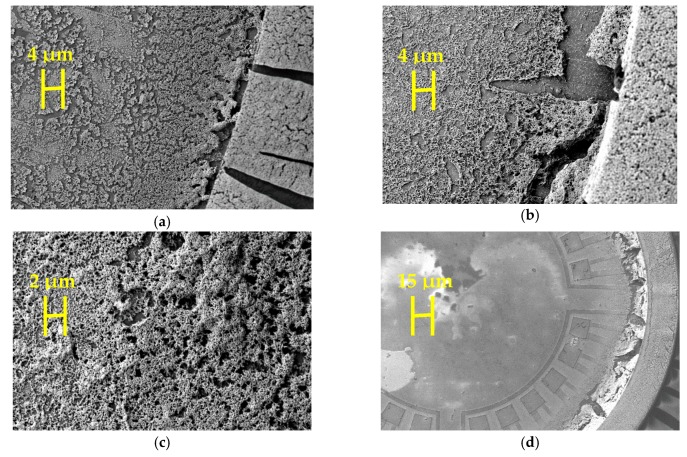

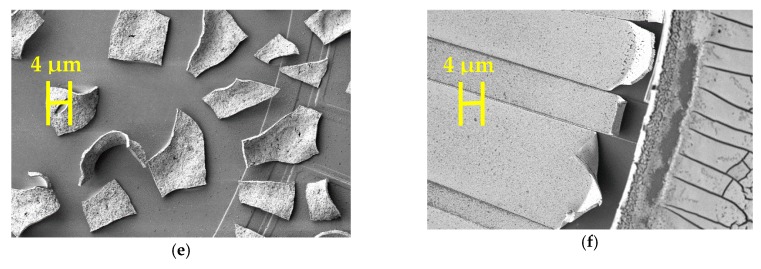
SEM image showing the condition of the catalyst after 72 h test (combination of three 24 h test at 130 °C, 110 °C and 90 °C operating temperature) at 1.5% continuous hydrogen flow for (**a**) Pt-PDA; (**b**) Pt-DATER; (**c**) PT-BEN; (**d**) Pt-DAN; (**e**) Pt-DACH; (**f**) Pt nanoparticles.

The SEM pictures in Figure 14a–f show the condition of six different catalysts after 72 h (three steps 24 h) test. The condition of Pt-PDA, Pt-DATER and Pt-BEN was the most stable. The porosity of the catalyst is still visible indicating less sintering among the nanoparticles. Some portions of the Pt-DAN catalysts have probably fallen off the active area during the test possibly due to bad adhesion to the substrate (also verified through an optical microscope). It seems that building mechanical stress in the layer of Pt-DACH was too high, therefore cracks were created in the layer and the layer peeled off. On the other hand, the cracks generated in the layer (Figure 14f) may indicate that a complete sintering of non-stabilized nanoparticles occurred in the experimental period. Cracks were also generated and the layer started to peel off.

Although different types of highly sensitive hydrogen gas sensors are reported in the literature, a sensor with long-term stability has yet to be demonstrated [12]. An improved stability of the catalytic layer has been achieved by our newly designed sensor with homogeneous temperature distribution. It was reported in a similar work that the signal of the sensor with Pt-PDA catalyst reduced 10% in 10 h when the sensor was tested in a continuous hydrogen flow of 1% at 130 °C operating temperature [24]. In contrast, the sensor presented in this work showed no degradation in sensor output for 24 h not only for Pt-PDA catalyst but also for other catalysts such as Pt-DATER, Pt-BEN, Pt-DAN, Pt-DACH when the sensor was tested at continuous hydrogen flow of even higher concentration in than previous work (1.5% at 130 °C operating temperature). Moreover, non-stabilized nanoparticles also showed better stability than in previously reported work [24,27]. The sensor shows linear characteristics with respect to hydrogen concentration. It can be said from the experimental results that the delamination of the catalyst such as Pt-DAN due to poor adhesion to the membrane material can be one possible reason for the deactivation. Additionally, the high stress created in the catalyst layer such as Pt-DACH during high-temperature operation can create cracks that can deactivate the catalyst. Surprisingly, almost no deviation in sensor signal for 24 h at low operating temperature (where catalyst deactivation is likely to occur due to water accumulation [27]), indicates that the sensor output is less affected by the generated water from hydrogen combustion reaction. Reproducibility of the sensor signal based on the reproducibility of the catalytic layer has yet to be investigated.

## 4. Conclusions

The main benefit of the proposed sensor is that it provides an excellent homogeneous temperature field on the membrane due to its optimized design that improves the stability of the ligand-linked nanoparticles catalyst. This paper has comprehensively discussed about the entire heat transfer criteria from the chip and its effect on homogeneous temperature field. This study has identified that the high convection heat loss coefficient, 217 W/m^2^ K, for the small chip is the reason for some inhomogeneity in the temperature field on the membrane. This is confirmed through mathematical calculation and real measurement of power consumption that exactly matched with each other. The most interesting finding is that the thermoelectric sensor consumes comparatively less power than a similar thermoresistive sensor for providing same average temperature on the membrane despite of having highly heat conductive thermopiles. The insights gained from the comparative stability analysis among six different catalyst, five different ligand-linked nanoparticles and unprotected nanoparticles, is beneficial for understanding the effect of different ligands on the stability of the catalyst during hydrogen gas sensing. Another major finding is that the sensor design also improves the stability of unprotected Pt nanoparticles.

## Figures and Tables

**Table 1:** List of the chemicals for catalyst preparation.

Chemicals	Purity (Weight %)	Company
hexachloroplatinic acid hydrate	(Pt-wt% 40.07)	ChemPur, Karlsruhe, Germany
ethylene glycol	99.7%	VWR International, Fontenay sous bois, France
ethanol	99.9%	VWR International, Fontenay sous bois, France
acetone	100.0%	VWR International, Fontenay sous bois, France
cyclohexanone	100.0%	VWR International, Fontenay sous bois, France
sodium hydroxide	99.3%	VWR International, Leuven, Belgium
*p*-phenylenediamine	97.0%	Alfa Aesar, Thermo Fisher Scientific, Kandel, Germany
benzidine	98.0%	Sigma-Aldrich, Steinheim, Germany
4,4′′-diamino-*p*-terphenyl	98.0%	TCI Europe, Zwijndrecht, Belgium
1,5-diaminonaphthalene	98.0%	TCI Europe, Zwijndrecht, Belgium
*trans*-1,4-diaminocyclohexane	98.0%	TCI Europe, Zwijndrecht, Belgium
